# Small but mighty: how can pediatric psychologists harness the power of digital single-session interventions?

**DOI:** 10.1093/jpepsy/jsag033

**Published:** 2026-08-01

**Authors:** Christina M. Amaro, Alexandra D. Monzon, Melissa A. Alderfer, Kimberly S. Canter, Michael Amylon, Stephanie Fooks-Parker, Jessica L. Schleider, Susana R. Patton, Katie A. Devine

**Affiliations:** 1 Rutgers Cancer Institute, New Brunswick, NJ, United States; 2 Center for Healthcare Delivery Science, Nemours Children’s Health, Orlando, FL, United States; 3 Center for Healthcare Delivery Science, Nemours Children’s Health, Wilmington, DE, United States; 4 Department of Pediatrics, Sidney Kimmel Medical College at Thomas Jefferson University, Philadelphia, PA, United States; 5 Division of Hematology/Oncology/Stem Cell Transplantation and Regenerative Medicine, School of Medicine, Stanford University, Stanford, CA, United States; 6 Division of Oncology, The Children’s Hospital of Philadelphia, Philadelphia, PA, United States; 7 Department of Medical Social Sciences, Feinberg School of Medicine, Northwestern University, Chicago, IL, United States; 8 Center for Healthcare Delivery Science, Nemours Children’s Health, Jacksonville, FL, United States

**Keywords:** digital interventions, mental health, psychosocial functioning, chronic illness, cancer, diabetes, adolescents, siblings

## Abstract

**Objective:**

Single-session interventions (SSIs) are an innovative, scalable approach for addressing the unmet behavioral and mental health needs of pediatric patients and their families. Specifically, digital SSIs, which are self-guided and delivered online, offer a low-cost and accessible approach but are currently underutilized in pediatric psychology.

**Methods:**

This topical review aims to describe SSIs broadly and the specific need for digital SSIs, explore potential applications within pediatric psychology, outline future directions and considerations, and present two examples of community-engaged digital SSI design.

**Results:**

SSIs evidence small to medium effects across a range of mental health concerns, including distress, anxiety, and depression, supporting their broad applicability. Incorporating community voices during the development process is essential to ensure SSIs are contextually relevant in pediatric populations. Two illustrative SSIs currently in development within pediatric psychology are described: (1) an intervention supporting adolescent siblings of children with cancer and (2) a program targeting depression in adolescents with type 1 diabetes. Both examples incorporate input from key community members, underscoring the value of participatory design in enhancing intervention relevance and impact.

**Conclusions:**

SSIs offer a brief and scalable strategy to help address gaps in psychological care, particularly for youth and families with limited access to services. Pediatric psychologists are well positioned to develop, evaluate, and implement SSIs across diverse populations and intervention targets. Ensuring the effectiveness, equity, and sustainability of SSIs will require ongoing collaboration with community partners and integration across pediatric care settings.

Despite the high need for psychological services, there are pervasive challenges to the provision of mental health care, as approximately 70% of youths do not access needed support (Mei et al., 2020). The demand for services has increased since the COVID-19 pandemic (Racine et al., 2021), but waitlists for outpatient therapy are long, and symptoms usually persist while waiting for services (Steinert et al., 2017). Staffing shortages exacerbate this problem (Anderson et al., 2017), as the limited number of pediatric psychologists available in specialty care clinics is unable to fully meet the high demand for support needs (Price et al., 2023).

Comprehensive, system-level strategies are essential to overcoming these persistent obstacles in traditional care models, and evidence-based digital interventions emphasizing accessibility may be one possible solution (Ghosh et al., 2023; Psihogios et al., 2024). Digital single-session interventions (SSIs), which are self-guided and delivered online, have emerged as a promising innovation, offering brief, scalable approaches that deliver targeted support in a single encounter; however, these remain underutilized in pediatric psychology despite their wide applicability for addressing common issues in the field. This topical review aims to describe SSIs broadly and the specific need for digital SSIs, explore potential applications within pediatric psychology, outline future directions and considerations, and present two examples of community-engaged digital SSI design.

## SSIs and the existing evidence base

SSIs are “specific, structured programs that *intentionally* involve just *one visit or encounter* with a clinic, provider, or program” (Schleider et al., 2020: 265). Given their deliberate brevity, SSIs draw from longer-term, multisession interventions by focusing on a single evidence-based strategy for presenting concerns, such as behavioral activation or cognitive appraisals for depression. However, SSIs are not limited to one type of theoretical orientation and can draw from various theoretical approaches, such as solution-focused and cognitive-behavioral techniques (Ghosh et al., 2023).

These brief, low-cost, and low-intensity interventions offer an alternative model of care to traditional psychotherapy, which is often time- and resource-intensive. While traditional psychotherapy can be beneficial for some, it does not sufficiently meet the needs of all young people and their families. In both community (Hoyt et al., 2018) and specialty care settings (Markowitz et al., 2014), multisession treatment is frequently terminated prematurely, with most individuals attending just one session. SSIs present a valuable opportunity to deliver therapeutic benefits to youth who may engage in only one session or who would otherwise not have access to traditional evidence-based services. The SSI model does not impose limits on the number of sessions required; instead, it accommodates varying needs and preferences. In this regard, SSIs share certain similarities with consultation models commonly used by pediatric psychologists to provide brief psychological services for youth and their families (Roberts et al., 2020). Both approaches can involve time-limited encounters and may draw on evidence-based strategies to support families in acute or resource-constrained contexts. At the same time, there are key conceptual and methodological differences between SSIs and consultation models. Although some pediatric consultation contexts operate under the practical reality that a given encounter may be the family’s only appointment, this assumption is not always made explicit to patients and is often not a defining feature of the model. In contrast, SSIs are informed by single-session thinking, which *intentionally* centers the premises that any clinical encounter might be a patient’s final engagement with therapy *and* that even a single session can be strategically leveraged to promote clinically meaningful change (Dryden, 2020; Hoyt, 2024). Consult models also vary widely in structure and scope depending on clinical context and may integrate multiple evidence-based elements or emphasize follow-up of resources and care coordination. Evidence-based SSIs, by comparison, typically employ a highly specified structure and focus on delivering a targeted intervention strategy within a single encounter, with explicit attention to within-session change processes. Moreover, pediatric consultation services often involve a variable number of encounters, with some families receiving one session and others receiving multiple visits. While this flexibility represents a core strength of consultation models, it can make it difficult to isolate the specific effects of single-session encounters. As such, evaluations of pediatric consultation services have largely emphasized outcomes such as referral completion or engagement in subsequent services rather than short-term changes in child mental health symptoms (e.g., Hurst et al., 2025). Thus, although SSIs and pediatric consultation models overlap in function and may occasionally converge in form, differences in intentionality, structure, and evaluation have resulted in largely parallel literatures to date. The current evidence base, therefore, does not yet allow this literature to be integrated cleanly, underscoring both the distinct theoretical and methodological contributions of SSIs within pediatric psychology and important opportunities for future research to more directly examine outcomes across these complementary models of care.

Evidence from an umbrella review of 415 unique trials synthesized across 24 systematic reviews published in the past two decades indicates that SSIs yield small to medium statistically significant effects across a range of mental health concerns, including distress, anxiety, depression, specific phobias, negative body image, and externalizing problems (Schleider et al., 2025). Among the 12 meta-analyses that directly compared SSIs to control conditions, findings revealed a small, positive overall effect across various problem types and age groups (*g* = −0.25). Although some in-person SSIs have been developed for youth with chronic conditions and their families (e.g., neurological disorders; Mulligan et al., 2022), none of the reviews included in the umbrella review focused on youth with chronic health conditions, and only one included meta-analysis that examined adults with chronic health conditions (Dochat et al., 2021). This gap highlights substantial opportunities to expand research in this area, particularly for youth with chronic health conditions and their families.

## The need for digital SSIs

Although SSIs can be delivered in various formats, including therapist-administered in-person or via telehealth, there is a growing need for digital SSIs delivered through online platforms and designed to be self-guided (i.e., self-administered without therapist involvement). Indeed, most U.S. households have access to smartphones and/or high-speed internet (Gelles-Watnick, 2024), and youths are natives of the digital world, making them particularly well-suited consumers of digital SSIs. Youth from minoritized backgrounds face additional barriers to treatment access, including stigma and provider mistrust, which contribute to service underutilization (Planey et al., 2019). However, seeking mental health information or help online provides a nonstigmatizing space for many adolescents and young adults (Pretorius et al., 2019). Parental consent can also be a barrier to traditional psychotherapy; yet, free digital self-guided SSIs can be safely accessed by youths without a parent present (Schleider et al., 2022). Furthermore, SSIs offer an evidence-based alternative to generative artificial intelligence tools in mental health, many of which remain largely untested and pose ethical and safety concerns (Wang et al., 2025). While digital SSIs are not intended to replace traditional service delivery models, they can be scalable options to bridge gaps in psychosocial care.

The umbrella review by Schleider and colleagues (2025) found that self-guided SSIs, which were delivered via digital or print formats, yielded improvements in at least one clinical domain for youth when compared to control groups. Self-guided and provider-delivered SSIs were shown to have equivalent effectiveness for youth (Schleider & Weisz, 2017), underscoring the versatility of these approaches and the potential benefits of using a digital format to enhance reach. Digital SSIs have demonstrated acceptability and feasibility among youth across diverse contexts, and these interventions have been successfully deployed in a variety of settings, including through research, schools, and social media platforms (Ghosh et al. 2023). Digital SSIs often use self-determination theory, which posits that proximal changes in relatedness, competence, and autonomy facilitate adaptive behavior change (Schleider et al., 2020). RCTs of SSIs suggest that these short-term changes (e.g., increasing hope, perceived control, and agency) are likely appropriate targets for SSIs, as they predict long-term reduction of psychological distress (Schleider et al., 2019).

## Applying the digital SSI approach within pediatric psychology

Logistical challenges, such as limited availability for weekly sessions in hospital settings and staffing shortages, often prevent pediatric psychologists from fully meeting the psychosocial needs of youth and families (Vassilopoulos et al., 2020). Digital SSIs offer a promising solution by increasing accessibility and facilitating scalable delivery of mental healthcare and, within the Pediatric Psychosocial Preventative Health Model (Kazak, 2006), can be conceptualized as low-intensity, universal interventions appropriate for all pediatric patients and their families. For more than two decades, pediatric psychologists have also explored how technology can enhance health care delivery and support the well-being of children and their families (Psihogios et al., 2024). Given these efforts, pediatric psychologists are well-equipped to develop, evaluate, and disseminate digital SSIs.

To date, digital SSIs are underutilized in pediatric psychology settings. This may be due to limited awareness of relevant digital SSIs, lack of tailoring to the unique challenges of pediatric chronic conditions and family needs, or challenges in implementation and dissemination of existing SSIs. As a first step, pediatric psychologists can incorporate existing, well-established digital SSIs into their practice. For instance, Project Youth Empowerment and Support (Project YES) contains several evidence-based digital SSIs to support adolescents and young adults in areas such as engaging in behavioral activation, practicing cognitive restructuring, developing a safety plan, and managing minority stress. Data from large RCTs of Project YES suggest significant improvements in adolescents’ perceived agency and control, along with reductions in hopelessness and distress symptoms (e.g., Schleider et al., 2022; Shen et al., 2023). Findings also demonstrate effectiveness across diverse groups, regardless of marginalized identity, language, or age, suggesting that Project YES is accessible and broadly beneficial (McDanal et al., 2024; Shroff et al., 2023). Project YES is now freely and anonymously available, with each digital SSI being approximately 5–10 min in English and Spanish (https://www.tryprojectyes.org/lsmh/). Pediatric psychologists can offer these resources to individuals on the waitlist and/or to existing patients as a way of complementing psychotherapy sessions.

While Project YES addresses many relevant mental health concerns for pediatric populations broadly, existing SSIs are not designed to meet the unique challenges and needs of youth with chronic illnesses and their families. Pediatric psychologists can address this gap by tailoring digital SSIs for pediatric populations specifically. Some target areas ripe for digital SSIs may include developing coping skills in the context of chronic health conditions, supporting healthcare transition, reinforcing self-management skills, preparing for hospitalization, and promoting health behaviors. For instance, Project Sleep promotes sleep in adolescents and young adults (Hamilton et al., 2025), a cross-cutting concern for many pediatric groups. Digital SSIs may also be designed to address issues relevant for specific pediatric populations, such as developing an action plan for managing acute and chronic pain in youth with sickle cell disease, reinforcing engagement in treatment for obstructive sleep apnea, or managing procedure-specific anxiety symptoms. Youth with chronic conditions and their families would likely benefit from a combination of transdiagnostic and chronic illness-specific digital SSIs.

Importantly, digital SSIs may fill gaps in pediatric psychology by potentially meeting the needs of the larger family system, including parents/caregivers and siblings, who are frequently affected by pediatric chronic health conditions but may not receive psychosocial supports. Prior work has also targeted digital SSIs to a professional audience, including addressing burnout in psychology trainees (Last et al., 2025), and there are avenues for expanding this work with psychologists and other healthcare team members. While there are opportunities to implement SSIs within pediatric medical settings (integrated primary care, inpatient, and outpatient clinics), this approach also appears ripe for expanding reach through other relevant contexts, such as school and community settings (Table 1).

## Digital SSI design and dissemination considerations within pediatric psychology

Despite the potential benefits of digital SSIs, there are limitations of this approach that should be considered. Digital SSIs are not designed to replace traditional psychotherapy services and are not substitutes for long-term therapies or higher levels of care. This approach may also not be ideal for every individual or every issue. SSIs based solely on psychoeducation have shown limited effectiveness, and incorporating active skill-building elements may be more impactful by fostering shifts in hope, self-efficacy, and perceived control (Schleider et al., 2025). Furthermore, similar to digital interventions more broadly, digital SSIs face challenges in moving interventions from research to real-world settings (Psihogios et al., 2024).

Given the purposeful brevity of SSIs, these interventions must be focused and cannot include all of the components of evidence-based treatments. As such, the research team must carefully choose the evidence-based treatment and standalone therapeutic component to target. Manvelian et al. (2025) provide a useful guide and checklist for adapting digital SSIs, including a description of the B.E.S.T.T. Principles used to enhance engagement and learning (i.e., *B*rain science to provide evidence in explaining key program concepts and normalize experiences; *E*mpowering the audience in their role as experts; *S*aying-is-believing exercises to facilitate learning of program concepts; *T*estimonials provided by program completers to reinforce core concepts; and *T*argeted by keeping the SSI brief and focused).

Digital SSIs offer many advantages to potential scale and dissemination, as they are often designed and tested in low-cost survey creation platforms, such as Qualtrics or REDCap (Manvelian et al., 2025). These platforms, widely used by universities, also permit researchers to advance digital SSIs through the intervention development pipeline more efficiently and align well with implementation-oriented frameworks such as the Accelerated Creation-to-Sustainment Model (Mohr et al., 2017), which aim to develop rigorous, evidence-based interventions while reducing dissemination delays (Psihogios et al., 2024). Examining existing workflows can also help to sustainably incorporate evidence-based digital SSIs. For instance, digital SSIs could be offered to those on a waitlist for psychotherapy services in conjunction with ongoing services, such as additional support after a one-time consultation with a pediatric psychologist, or as a standalone universal program. Co-production with individuals who would be using and disseminating the digital SSI is critical to ensuring these interventions are contextually relevant and responsive to the needs of the population they intend to serve (Manvelian et al., 2025; Psihogios et al., 2024).

There are several future directions for digital SSIs in pediatric psychology research and practice. Although possible applications are described in this topical review, community-engaged research is needed to determine if these are appropriate targets and to explore additional avenues for youth with chronic conditions and their families. Existing systematic reviews of SSIs focus on mental health and service engagement, and there are opportunities to explore behavioral health outcomes. In addition to examining short- and long-term treatment outcomes, it is important to examine facilitators and barriers to the dissemination and implementation of digital SSIs within pediatric psychology settings. Quality improvement projects may be useful to gain feedback from clinicians and families on how well SSIs are integrated into care and if they experience clinically meaningful changes. By thoughtfully addressing design, dissemination, and community collaboration, digital SSIs can be powerful tools for expanding access to evidence-based care within pediatric psychology, especially for underserved populations.

## Two examples of digital SSI applications in pediatric psychology

The following two examples illustrate applications of digital SSIs within pediatric psychology, demonstrating how a community-engaged approach can inform the development and adaptation of SSIs for specific pediatric populations.^1^

### An SSI to support adolescent siblings of youth with cancer

Supporting siblings is a Psychosocial Standard of Care in Childhood Cancer (Gerhardt et al., 2015). However, several barriers limit services for siblings, including siblings’ frequent absence from the hospital setting, staff shortages, and few evidence-based interventions tailored to siblings (Brosnan et al., 2022). This includes a relative lack of digital health interventions (Mooney-Doyle et al., 2021). Digital SSIs have the potential to overcome barriers and address this gap in sibling psychosocial care. Researchers first gathered qualitative data regarding siblings’ perspectives on digital health interventions and a digital SSI specifically. The team also gathered input from parents, psychosocial providers, and community organizations regarding their unique insights into psychosocial service delivery for families (Amaro et al., 2026).

Adolescent siblings (*n* =22) and parents (*n* = 21) of children with cancer and professionals (*n* =14) participated in the qualitative study (Supplementary Table S1). Participants noted several advantages of digital health interventions, including the potential to overcome barriers and increase sibling service access (Supplementary Table S2). Digital SSIs were seen as a valuable, brief resource that could convey to siblings that their well-being matters and help them feel included. Concerns were also raised, including limited access to internet/devices or the SSI being too generic. While a sibling SSI may not address every sibling’s concerns, participants suggested that siblings could be provided a list of additional resources at the end of the SSI. Overall, community members viewed a digital SSI for siblings as a meaningful approach with broad applicability, emphasizing its potential to support sibling well-being. Ongoing research is adapting a solution-focused SSI for adolescent siblings of children with cancer.

### An SSI to reduce depression and HbA1c in adolescents with type 1 diabetes

Managing type 1 diabetes (T1D) involves a complex daily regimen of monitoring carbohydrate intake and glucose levels, administering insulin, and engaging in physical activity (American Diabetes Association Professional Practice Committee, 2025). Unfortunately, most youth under 18 years do not meet glycemic control targets, as measured by HbA1c, and data suggest that many youth and their families also struggle to manage T1D effectively (Foster et al., 2019). Further, meta-analyses suggest that up to 30% of youth with T1D experience elevated depressive symptoms (Akbarizadeh et al., 2022), which can complicate one’s ability to engage in T1D management. Most existing behavioral interventions to help youth with T1D are resource-intensive and are underutilized in clinical practice (Price et al., 2019).

A digital SSI that incorporates evidence-based strategies to reduce depressive symptoms and lower glucose levels could fill a care gap for youth with T1D. To do so, researchers on the Adapting Single Sessions Interventions for Type 1 Diabetes (ASSISTED) study partnered with a Youth Advisory Board (YAB) comprised of eight youth aged 12–17 years to adapt an existing depression SSI for youth with T1D. YAB members agreed that a depression SSI specific to youth with T1D likely meets a critical need, but the timing and delivery of this intervention require careful consideration, as many youths may not appreciate another service being added to their endocrinology visit. Next, the ASSISTED study team interviewed 5 diabetes providers and 10 youth with T1D to further inform the SSI adaptation. Youth described how low mood and depressive symptoms contribute to the avoidance of T1D tasks (i.e., administering insulin, changing pump sites) and how interpersonal stressors (e.g., family conflict and stigma) exacerbated this avoidance (Supplementary Table S3). Providers also noted that insulin administration seemed to be the most impacted by depressive symptoms. This ongoing study is in the process of adapting the digital depression SSI for youth with T1D for future acceptability and feasibility testing.

## Conclusion

SSIs broadly, and digital SSIs specifically, offer a promising, low-burden approach to addressing gaps in care, particularly for youth and families with limited access to psychological services. Pediatric psychologists are well positioned to advance the development, evaluation, and implementation of SSIs across diverse settings. Complementing existing psychotherapy services, digital SSIs may also be the only feasible option for some or a catalyst for others to pursue additional support. Engaging youth, families, and other relevant individuals in co-design and leveraging dissemination frameworks will be essential to ensuring SSIs are effective, equitable, and sustainably integrated into care.

## Supplementary Material

Supplemental materials

Supplementary material is available at *Journal of Pediatric Psychology* online.

## Figures and Tables

**Table 1:** Potential advantages and disadvantages of digital single-session interventions for pediatric psychology.

Potential advantages of digital SSIs	Potential disadvantages of digital SSIs

• SSIs are brief, low-cost, low-intensity interventions • Can increase accessibility, particularly for individuals from marginalized backgrounds, and facilitate scalability • While SSIs are intentionally designed for one session, they can be completed multiple times, as needed • SSIs may support family members who are unable to access support in the healthcare setting • Given the flexibility of SSIs, they can be delivered across settings (e.g., social media, community)	• SSIs are focused and cannot include all of the components of evidence-based treatments • SSIs are not tailored to meet the unique needs of each individual and may not be a good fit for each individual • SSIs do not eliminate the need for higher-intensity interventions • SSIs cannot overcome accessibility issues for those with limited or no access to the internet and/or electronic devices
**Key takeaways:**	
• Digital SSIs offer a promising, low-intensity approach to addressing gaps in psychosocial care, and are designed to complement, rather than replace, existing traditional, multisession therapies • Pediatric psychologists are well-equipped to advance the development, evaluation, and dissemination of digital SSIs across populations and settings	

*Note.* SSI = single-session intervention.

## Footnotes

1 De-identified data are available upon reasonable request.
