# Supplementary material for: Small but mighty: how can pediatric psychologists harness the power of digital single-session interventions?
Source: J Pediatr Psychol. 2026 May 8;51(8):717–23. doi: 10.1093/jpepsy/jsag033 (PMC13310525; doi:10.1093/jpepsy/jsag033)
Supplement: Supplementary file 1 [file jsag033_supplementary_data.zip › jsag033_Supplementary_Data/jpepsy-2025-0258-File003.docx]

Supplementary Table 1. Demographic Characteristics

| Characteristic | Siblings ^a^ (n=22)  n (%) | Parents (n=21)  n (%) | Professionals (n=14)  n (%) |
| --- | --- | --- | --- |
| Age (M, SD) | 14.9 (1.5) | 41.7 (4.8) | - |
| Gender |  |  |  |
| Female | 14 (63.6) | 18 (85.7) | 13 (92.9) |
| Male | 8 (36.4) | 3 (14.3) | 1 (7.1) |
| Race/Ethnicity ^b^ |  |  |  |
| White | 13 (59.1) | 11 (52.4) | 13 (92.9) |
| Latino/x or Hispanic | 7 (31.8) | 7 (33.3) | 1 (7.1) |
| Black | 2 (9.1) | 3 (14.3) | 0 |
| South Asian | 1 (4.5) | 1 (4.8) | 1 (7.1) |
| Asian | 2 (9.1) | 1 (4.8) | 0 |
| Middle Eastern | 2 (9.1) | 0 | 0 |
| Cancer Type of Diagnosed Child |  |  |  |
| Leukemia | – | 11 (52.4) | - |
| Solid, Bone, and Tissue Cancers | – | 4 (19.1) | - |
| Lymphoma | – | 3 (14.3) | - |
| Brain and CNS Tumors | – | 3 (14.3) | - |
| Professional Role |  |  |  |
| Psychologist | - | - | 6 (42.9) |
| Social Worker | - | - | 4 (28.6) |
| Child Life Specialist | - | - | 1 (7.1) |
| Art Therapist | - | - | 1 (7.1) |
| Director of Community Organization | - | - | 2 (14.3) |

*Note*. ^a^ Family members were eligible if they were a sibling (ages 13-17 years old) or parent of a child diagnosed with cancer and fluent in English or Spanish (three parents conducted interviews in Spanish). Professionals were eligible if they were psychosocial providers or community administrators in the US. ^b^ Totals may exceed 100%, as participants could check all that apply: Native American, American Indian, Indigenous, or Alaska Native (Navajo nation, Mayan, Aztec, Native Village or Barrow Inupiat Traditional Government, Nome Eskimo Community, etc.), Asian (Chinese, Filipino, Korean, Japanese, Vietnamese, etc.), South Asian (Pakistani, Indian, Sri Lankan, Bangladeshi), Southeast Asian (Cambodian, Laos, Thai), Black (Afro-Latino, African American, Haitian, Jamaican, Nigerian, Ethiopian etc.), Latino/x or Hispanic (from a Latin American country or Spanish origin/Spanish-speaking nation), Middle Eastern or North African (Lebanese, Iranian, Egyptian, Syrian, Moroccan, Algerian, etc.), Native Hawaiian or Other Pacific Islander (Samoan, Chamorro, Tongan, Fijian, etc.), White (German, English, Irish, French, Italian, Polish, etc.), and/or Not Listed.
