# Supplementary material for: Small but mighty: how can pediatric psychologists harness the power of digital single-session interventions?
Source: J Pediatr Psychol. 2026 May 8;51(8):717–23. doi: 10.1093/jpepsy/jsag033 (PMC13310525; doi:10.1093/jpepsy/jsag033)
Supplement: Supplementary file 1 [file jsag033_supplementary_data.zip › jsag033_Supplementary_Data/jpepsy-2025-0258-File005.docx]

Supplementary Table 3. Adolescent and Provider Perspectives on a Depression Intervention for Teens with Type 1 Diabetes

| Theme | Summary | Exemplar Quotes |
| --- | --- | --- |
| Impact of Depression on Diabetes Management | Teens and providers consistently described how low mood leads to reduced engagement in diabetes care, especially skipping insulin doses and ignoring devices. Depression generally reduces motivation and energy to engage in self-care. | "Sometimes I just stay in bed and don’t even check my blood sugar." – Teen  "They don’t have the motivation to do anything about their diabetes when feeling depressed." – Provider |
| Avoidance and Hopelessness | Avoidance behaviors are common, including skipping meals, ignoring alarms, or not bolusing. Teens often feel hopeless about managing diabetes when mood is low. | "Some teens forget to give insulin before they eat, so they just didn’t give it at all." – Provider  "Why bother checking? It’s going to be bad anyway." – Teen |
| Family Dynamics and Conflict | Parent-child conflict is a frequent barrier. Teens feel overwhelmed by responsibility; parents struggle with how much to let go. Providers often witness arguments in clinic. | "I’ve had kids and parents argue in front of me." – Provider  "My mom always ask if I did my bolus. They don’t have to do anything." – Teen |
| Overlap of Depression and Diabetes Symptoms | Teens and providers noted how depression and diabetes both affect sleep, eating, and concentration. High blood sugar worsens mood and physical symptoms. | "When my sugar’s high, I feel sick and don’t want to do anything." – Teen  "Their mood is entirely dependent on their blood sugars." – Provider |
| Intervention Preferences | Teens prefer short (30–50 min), flexible formats. Many favor online delivery but want it to be interactive. Providers suggest offering both in-person and telehealth options. | "Online is good, but I want someone to do it with me." – Teen  "If we find someone is depressed, we should prioritize meeting with a psychologist that day." – Provider |
| Timing and Feasibility | Teens prefer interventions separate from clinic visits. Providers worry about follow-through if not offered during appointments. Space and time constraints in clinic are major barriers. | "I don’t want another thing added to my appointment." – Teen  "You won’t get follow-through if it’s on another day." – Provider |
| Suggestions for Intervention Content | Include examples of diabetes-related avoidance, normalize burnout, and emphasize asking for help. Providers recommend integrating psychoeducation on how blood sugar affects mood. | "Receiving help doesn’t mean you’re failing." – Provider  "Add info on how to control blood sugar and what to do when it’s high." – Teen |
