# Supplementary material for: Small but mighty: how can pediatric psychologists harness the power of digital single-session interventions?
Source: J Pediatr Psychol. Author manuscript; Available in PMC 2026 Jun 28. (PMC13310525; doi:10.1093/jpepsy/jsag033)
Supplement: Supplemental materials [file NIHMS2184358-supplement-Supplemental_materials.docx]

Supplementary Table 1. Demographic Characteristics

| Characteristic | Siblings ^a^ (n=22)  n (%) | Parents (n=21)  n (%) | Professionals (n=14)  n (%) |
| --- | --- | --- | --- |
| Age (M, SD) | 14.9 (1.5) | 41.7 (4.8) | - |
| Gender |  |  |  |
| Female | 14 (63.6) | 18 (85.7) | 13 (92.9) |
| Male | 8 (36.4) | 3 (14.3) | 1 (7.1) |
| Race/Ethnicity ^b^ |  |  |  |
| White | 13 (59.1) | 11 (52.4) | 13 (92.9) |
| Latino/x or Hispanic | 7 (31.8) | 7 (33.3) | 1 (7.1) |
| Black | 2 (9.1) | 3 (14.3) | 0 |
| South Asian | 1 (4.5) | 1 (4.8) | 1 (7.1) |
| Asian | 2 (9.1) | 1 (4.8) | 0 |
| Middle Eastern | 2 (9.1) | 0 | 0 |
| Cancer Type of Diagnosed Child |  |  |  |
| Leukemia | – | 11 (52.4) | - |
| Solid, Bone, and Tissue Cancers | – | 4 (19.1) | - |
| Lymphoma | – | 3 (14.3) | - |
| Brain and CNS Tumors | – | 3 (14.3) | - |
| Professional Role |  |  |  |
| Psychologist | - | - | 6 (42.9) |
| Social Worker | - | - | 4 (28.6) |
| Child Life Specialist | - | - | 1 (7.1) |
| Art Therapist | - | - | 1 (7.1) |
| Director of Community Organization | - | - | 2 (14.3) |

*Note*. ^a^ Family members were eligible if they were a sibling (ages 13-17 years old) or parent of a child diagnosed with cancer and fluent in English or Spanish (three parents conducted interviews in Spanish). Professionals were eligible if they were psychosocial providers or community administrators in the US. ^b^ Totals may exceed 100%, as participants could check all that apply: Native American, American Indian, Indigenous, or Alaska Native (Navajo nation, Mayan, Aztec, Native Village or Barrow Inupiat Traditional Government, Nome Eskimo Community, etc.), Asian (Chinese, Filipino, Korean, Japanese, Vietnamese, etc.), South Asian (Pakistani, Indian, Sri Lankan, Bangladeshi), Southeast Asian (Cambodian, Laos, Thai), Black (Afro-Latino, African American, Haitian, Jamaican, Nigerian, Ethiopian etc.), Latino/x or Hispanic (from a Latin American country or Spanish origin/Spanish-speaking nation), Middle Eastern or North African (Lebanese, Iranian, Egyptian, Syrian, Moroccan, Algerian, etc.), Native Hawaiian or Other Pacific Islander (Samoan, Chamorro, Tongan, Fijian, etc.), White (German, English, Irish, French, Italian, Polish, etc.), and/or Not Listed.

Supplementary Table 2. Community Perspectives of Digital Health and Single-Session Interventions for Adolescent Siblings of Children with Cancer

| **Theme** ^a^ | **Summary** | **Exemplar Quotes** |
| --- | --- | --- |
| Potential Benefits of Digital Health Interventions | -Providing a space for siblings  -Increase access by overcoming barriers like transportation, scheduling, and limited staffing  -Offers flexible, convenient, and targeted support in a format that adolescents widely utilize  -Reduces mental health stigma and provides immediate help without needing a therapist first | “I think it’s a good idea, just because people have the privacy of being in their home. I think that kind of gives them a security blanket in a way. So, yeah, specifically digitally, it’s probably just for some people they might be more comfortable being on a computer screen, rather than being in person.” -17-year-old female sibling #142  “Well, it's very appealing in the sense that it's easily accessible. You know, like if you need something, you can just open a computer and it can be right there versus trying to have to set up an appointment with a mental health professional, say to go like talk to someone. And especially as a parent of a sick child, like so much of my life just revolved around not only [child with cancer]'s care, but also like her treatment…It was very overwhelming. So, I think the idea of having a digital access to something is very appealing." -Mother #424  “So, ease of access is a big one. And then I think also just being able to provide resources to a large number of teens in a relatively quick way, I think is probably the best way to summarize it.” -Female psychosocial provider #208 |
| Potential Benefits of Single-Session Interventions | -Self-paced and digital format can easily fit into adolescents’ busy schedules  -Short, manageable, and low barriers, as there is no long-term commitment  -Validates siblings experiences, offering them coping tools and focusing on their unique needs  -Has potential to bridge to other supports and potentially foster family communication | “And I think the part where you said that it was self-guided– that part definitely stood out to me because – like I take my – a lot of time on things, so I'd like to go at my own pace and my own speed, just kind of like – you know, like just kind of like do it by myself at like my own pace and not like being rushed or anything.” -15-year-old female sibling #110B  “It’s kind of short, sweet, to the point and a single session could be attainable and could feel attainable and not feel cumbersome to the child.” -Mother #442  “I would love [a Sibling SSI] in our system. I would love to refer families to something like that…I feel like this would be the most popular at my institution. When we identify an issue, and we’re like, here’s a 30-minute or less session that we’d love for you to see that would talk directly to what you’re dealing with.” -Female psychosocial provider #201 |
| Potential Disadvantages of Digital Health Interventions | -Lacks in-person connection and direct interaction with a therapist  -Not a good fit for all siblings, as some many need more structure, guidance, or individualized support  -Challenges with access, privacy, and engagement; risk of feeling generic  -Needs to be optional, visually appealing, and thoughtfully designed to hold interest | “I'm not sure if the digital programs would be that effective. There's a lot of similar resources. When [the cancer diagnosis] happened, I did try to research stuff on my own. But I feel like most of the stuff was general advice…” -17 year-old female sibling #128  “I don't want her on the internet by herself. That - she's mature enough to handle it, but our other - you know, our boys are adopted and they have a lot of just impulse control issues, and so it would need to be in a supportive way that they could access them…Another concern I would have, it's not an issue for our family, but I do work at a school where you're assuming people have internet, laptop, computer access, and many people don't have that at home, so are they going to go to the library and access it, or how are we going to make sure that they can - that it's not only being available to those who have the means to have the things you would need to be at?” -Mother #489  “But, you know, it's one of those things where it's like, it's either great for somebody that they're like gonna be a high utilizer and really adopt it and it's like the best thing ever, or it's like that person is craving something else and then it's just not gonna work…But what I would like to see like ideally is that those things, whatever digital stuff is offered is always more of an adjunct to formal care or a, you know, like something that comes before, right? That like you do that as like bridging therapy per se until you can get to the real deal kind of thing. Because I think that there is no real substitute for true psychotherapy.” -Female psychosocial provider #208 |
| Potential Disadvantages of Single-Session Interventions | -Some siblings will require more structured, ongoing support beyond a single session  -Engagement may be low if content does not feel personally relevant or compelling  -Without a social component, siblings do not have an opportunity to directly connect with other siblings  -Without follow up, siblings may not fully understand or expand on the material | “…. sometimes maybe more time is needed... It’s a very complex topic, you know, obviously. It’s a very hard scenario that they’re going through – or that we’re going through. So, I think more than [a single session] could be needed for some siblings.” -13-year-old male sibling #107  “Digital [SSIs are] easy, they’re used to [digital programs] in school and everything else, so it’s convenient, it’s quick. The only concern would be that it opens up something…what do they do next, it’s a single session.” -Father #428  “… for our siblings that have higher needs, I think this [Sibling SSI] is still helpful and it's beneficial, but it likely will not be the end-all be-all for them for treatment…So, just making sure for those siblings, if it is like more of like a triage, you're going to need additional support and making sure they get connected with resources, that [a Sibling SSI] is an initial step. And then there's other avenues they can go, I think is an important component.” -Female psychosocial provider #202 |

Note. ^a^ Semi-structured interviews were conducted individually with each participant in English or Spanish from October 2023 through January 2025 through Zoom. Interviews were transcribed verbatim, deidentified, cleaned for accuracy, and summarized to distill findings. Three parents completed interviews in Spanish; interviews were translated to English for qualitative coding and analysis.

Supplementary Table 3. Adolescent and Provider Perspectives on a Depression Intervention for Teens with Type 1 Diabetes

| Theme | Summary | Exemplar Quotes |
| --- | --- | --- |
| Impact of Depression on Diabetes Management | Teens and providers consistently described how low mood leads to reduced engagement in diabetes care, especially skipping insulin doses and ignoring devices. Depression generally reduces motivation and energy to engage in self-care. | "Sometimes I just stay in bed and don’t even check my blood sugar." – Teen  "They don’t have the motivation to do anything about their diabetes when feeling depressed." – Provider |
| Avoidance and Hopelessness | Avoidance behaviors are common, including skipping meals, ignoring alarms, or not bolusing. Teens often feel hopeless about managing diabetes when mood is low. | "Some teens forget to give insulin before they eat, so they just didn’t give it at all." – Provider  "Why bother checking? It’s going to be bad anyway." – Teen |
| Family Dynamics and Conflict | Parent-child conflict is a frequent barrier. Teens feel overwhelmed by responsibility; parents struggle with how much to let go. Providers often witness arguments in clinic. | "I’ve had kids and parents argue in front of me." – Provider  "My mom always ask if I did my bolus. They don’t have to do anything." – Teen |
| Overlap of Depression and Diabetes Symptoms | Teens and providers noted how depression and diabetes both affect sleep, eating, and concentration. High blood sugar worsens mood and physical symptoms. | "When my sugar’s high, I feel sick and don’t want to do anything." – Teen  "Their mood is entirely dependent on their blood sugars." – Provider |
| Intervention Preferences | Teens prefer short (30–50 min), flexible formats. Many favor online delivery but want it to be interactive. Providers suggest offering both in-person and telehealth options. | "Online is good, but I want someone to do it with me." – Teen  "If we find someone is depressed, we should prioritize meeting with a psychologist that day." – Provider |
| Timing and Feasibility | Teens prefer interventions separate from clinic visits. Providers worry about follow-through if not offered during appointments. Space and time constraints in clinic are major barriers. | "I don’t want another thing added to my appointment." – Teen  "You won’t get follow-through if it’s on another day." – Provider |
| Suggestions for Intervention Content | Include examples of diabetes-related avoidance, normalize burnout, and emphasize asking for help. Providers recommend integrating psychoeducation on how blood sugar affects mood. | "Receiving help doesn’t mean you’re failing." – Provider  "Add info on how to control blood sugar and what to do when it’s high." – Teen |
